# Enhanced Photocatalytic Activity of the Carbon Quantum Dot-Modified BiOI Microsphere

**DOI:** 10.1186/s11671-016-1262-7

**Published:** 2016-02-03

**Authors:** Yuan Chen, Qiuju Lu, Xuelian Yan, Qionghua Mo, Yun Chen, Bitao Liu, Liumei Teng, Wei Xiao, Liangsheng Ge, Qinyi Wang

**Affiliations:** Research Institute for New Materials Technology, Chongqing University of Arts and Sciences, Yongchuan, Chongqing, 402160 China; School of Materials Science and Engineering, Chongqing University of Technology, Banan, Chongqing, 400054 China; Faculty of Materials and Energy, Southwest University, Beibei, Chongqing, 400715 China; Department of Chemical Engineering, University of Missouri, Columbia, MO 65211-2200 USA

**Keywords:** BiOI, CQDs, Photocatalytic, Visible light

## Abstract

Novel carbon quantum dot (CQD)-modified BiOI photocatalysts were prepared via a facile hydrothermal process. The CQD-modified BiOI materials were characterized by multiple techniques. The CQD with an average size around several nanometers was distributed on the surface of BiOI microsphere. Its photocatalytic activity was investigated sufficiently by the photodegradation of methylene orange (MO). The results showed that the CQD/BiOI 1.5 wt.% sample exhibited the optimum photocatalytic activity, which was 2.5 times that of the pure BiOI. This improvement was attributed to the crucial role of CQDs, which could be acted as a photocenter for absorbing solar light, charge separation center for suppressing charge recombination.

**Electronic supplementary material:**

The online version of this article (doi:10.1186/s11671-016-1262-7) contains supplementary material, which is available to authorized users.

## Background

The exploration and construction of new photocatalysts with high catalytic efficiency in sunlight is a core issue in photocatalysis all the time and is also significant in solving current environment and energy problems [1–3]. Recently, bismuth oxyhalides (BiOX, *X* = Cl, Br, and I) as a novel ternary oxide semiconductor have drawn much attention because of their potential application in photocatalysis. Among them, BiOI is photochemically stable and has the smallest band gap (about 1.7–1.9 eV), which can be activated by visible light irradiation [4–6]. However, the narrow band gap could also lead to a quick recombination of the photogenerated electron–hole pairs. Hence, inhibiting the recombination of the photogenerated electron–hole pairs was the key point to enhance the photocatalytic property.

Carbon quantum dot (CQD), as a novel issue of recently found nanocarbons, exhibits excellent photophysical properties. Especially, the strong size and excitation wavelength-dependent photoluminescence (PL) behaviors would enhance the photocatalytic properties of the CQD-based composites [7, 8]. Previous studies have shown that the electron-accepting and transport properties of carbon nanomaterials provide a convenient way to separate photogenerated electrons; thus, enhanced photocatalytic performance can be achieved through the construction of semiconductor/carbon composites [9, 10]. Notably, the design of complex photocatalysts (TiO_2_/CQDs, Ag_3_PO_4_/CQDs, Bi_2_MoO_6_/CQDs) to utilize more sunlight has been reported [11–13]. Considering such remarkable properties of CQDs and the limitations of the BiOI photocatalytic system, the combination of CQDs and BiOI may be regarded as an ideal strategy to construct highly efficient complex photocatalytic systems.

In this work, we prepared a CQD/BiOI nanocomposite photocatalyst via a facile hydrothermal process. The results indicated that the CQDs were successfully combined with the BiOI microsphere and the introduction of CQDs could efficiently increase the concentration and the migration ratio of the photogenerated carrier, which was the key for the increased photocatalytic property.

## Methods

### Reagents

All chemicals used in this study were of analytical grade (ChengDu Kelong Chemical Co.) and were used without further purification. Citric acid (C_6_H_8_O_7_ · H_2_O, 99.5 %), ethylenediamine (C_2_H_8_N_2_, 99 %), Bi(NO_3_)_3_ · _5_H_2_O (99 %), KI (99 %), ethylene glycol (C_2_H_6_O_2_, 99.5 %), ethanol (C_2_H_6_O, 99.7 %), and distilled water were used in all experiments.

### Synthesis of CQD-Modified BiOI

CQD powder was synthesized according to the literature followed by freeze drying [14]. BiOI microspheres were synthesized by a facile solvothermal method. Typically, 0.4 g KI and 1.16 g Bi(NO_3_)_3_ · 5H_2_O were dissolved in 40 mL of ethylene glycol. Then, a certain content of CQD powder was added into the solution. Subsequently, the mixture was transferred to a 50-mL Teflon-lined stainless steel autoclave and the reaction was kept at 160 °C for 12 h. Finally, the resulting precipitate was collected, washed thoroughly with deionized water and ethanol, and dried at 60 °C in vacuum. Pure BiOI and CQD-modified BiOI samples with different mass ratios (0.5, 1, 1.5, and 2 wt.%) were synthesized using a similar route by tuning the content of CQDs.

### Instruments

The X-ray diffraction (XRD) patterns of the samples were recorded on a Danton TD-3500 X-ray diffractometer using Cu Kα radiation (*λ* = 1.54 Å). The field-emission scanning electron microscopy (FE-SEM) measurements were carried out with a field-emission scanning electron microscope (Hitachi, SU-8020). Transmission electron microscopy (TEM) micrographs were taken with a JEOL-JEM-2010 (JEOL, Japan) operated at 200 kV. Fourier transform infrared (FT-IR) spectra (KBr pellets) were recorded on Nicolet model Nexus 470 FT-IR equipment. X-ray photoelectron spectroscopy (XPS) analysis was performed on an ESCA Lab MKII X-ray photoelectron spectrometer using the Mg Kα radiation. UV-vis absorption spectra of the samples were obtained on a UV-vis spectrophotometer (Hitachi, U-3900), and BaSO_4_ powder was used as the substrate. The PL spectra were measured using a customized single-photon counting system (Beijing Zolix), A He-Ga laser (*λ* = 325 nm) was used as the excitation source. The photoelectric performance was measured using an electrochemical system (CHI-660B, China). BiOI and CQD/BiOI electrodes served as the working electrode; the counter and the reference electrodes were a platinum wire and a saturated Ag/AgCl electrode, respectively. A solution of 0.1 M NaSO_4_ was used as an electrolyte solution for the measurement, and a 150-W Xe arc lamp was utilized as the light source for the photoelectrochemical (PEC) measurement. The photoresponse of the photocatalysts in the presence and absence of visible light was measured at 0.0 V. Electrochemical impedance spectra (EIS) were recorded in the open circuit potential mode, and the frequency was ranged from 100 kHz to 0.01 Hz.

### Trapping Experiment

Potassium iodide (KI), tertbutyl alcohol (TBA), and potassium dichromate (K_2_Cr_2_O_7_) were used to trap hole, ·OH, and photogenerated electrons, respectively. Photocatalyst (0.1 g) with different trapping agents was added into MO (100 mL, 50 mg/L) aqueous solution. The scavengers used in this research are tertbutyl alcohol (TBA, 1 %) for ·OH, potassium dichromate (K_2_Cr_2_O_7_, 1 %) for e^−^, and potassium iodide (KI, 1 %) for h^+^, respectively.

### Photocatalytic Activity Measurement

The photocatalytic activities of the as-prepared samples were evaluated by the degradation of methyl orange (MO) under visible light irradiation at ambient temperature using a 150-W Xe lamp with a 420-nm cutoff filter as the light source. In each experiment, 100 mg of photocatalyst was dispersed in an MO (100 mL, 50 mg L^−1^) aqueous solution. Prior to irradiation, the solution was continuously stirred in the dark for 1 h to ensure the establishment of adsorption–desorption equilibrium between the photocatalysts and the degrading pollutants. During the photoreactions, the MO solutions with photocatalysts were continuously stirred with magnetometric stirrer, and a 3-mL sample solution was taken out at every 10-min interval during the experiment, followed by centrifugation and filtration to remove the photocatalysts. The concentrations of MO were determined by monitoring the change of optical density at 465 nm, with a Varian UV-vis spectrophotometer (Cary-50, Varian Co.).

## Results and Discussion

**Fig. 1:**
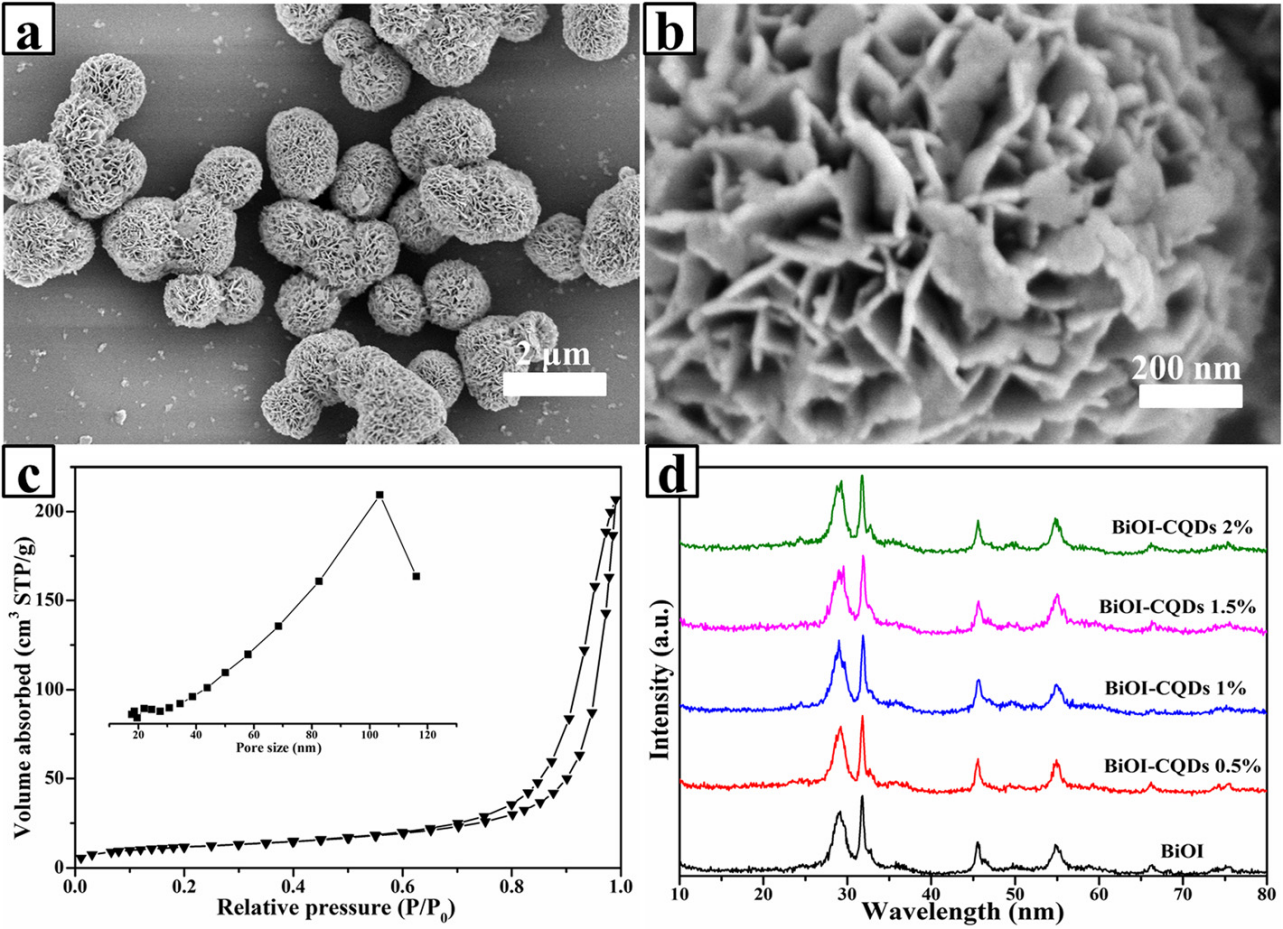
The SEM images (**a**, **b**) and nitrogen adsorption–desorption isotherms. Insert: the corresponding pore size distributions of CQD/BiOI 1.5 wt.% sample (**c**). XRD patterns of the series of CQD/BiOI composites (**d**)

The morphology of the as-prepared CQD/BiOI composites was shown in Fig. 1a, b. As seen, the sample was composed of uniform layered structure nanoplates and presented microsphere morphology. The diameter was about 1 to 2 μm and the thickness of the nanoplates was less than 50 nm. The SEM images of the other series samples were also given in Additional file 1: Figure S1, and it can be seen that the adding of CQDs would not change the original morphologies of BiOI. The nitrogen adsorption–desorption isotherms and the corresponding pore size distributions of the as-obtained samples were shown in Fig. 1c. According to the result, the calculated specific surface area was 42 m^2^/g. Obviously, this large specific surface area could have a positive effect on photocatalytic property [15, 16].

The XRD patterns of the series of CQD/BiOI composites were shown in Fig. 1d. It can be clearly seen that these photocatalysts were crystallized in a single phase. All the samples can be indexed to the tetragonal structure BiOI (JCPDS 10-0445). However, for the CQD-modified BiOI samples, no characteristic peak of CQDs (about 26°) can be found, which should be attributed to the low CQD content in the samples. Actually, if the content was lower than 5 %, which was hardly characterized by XRD, similar work was also demonstrated in the previous report [12, 17].

**Fig. 2:**
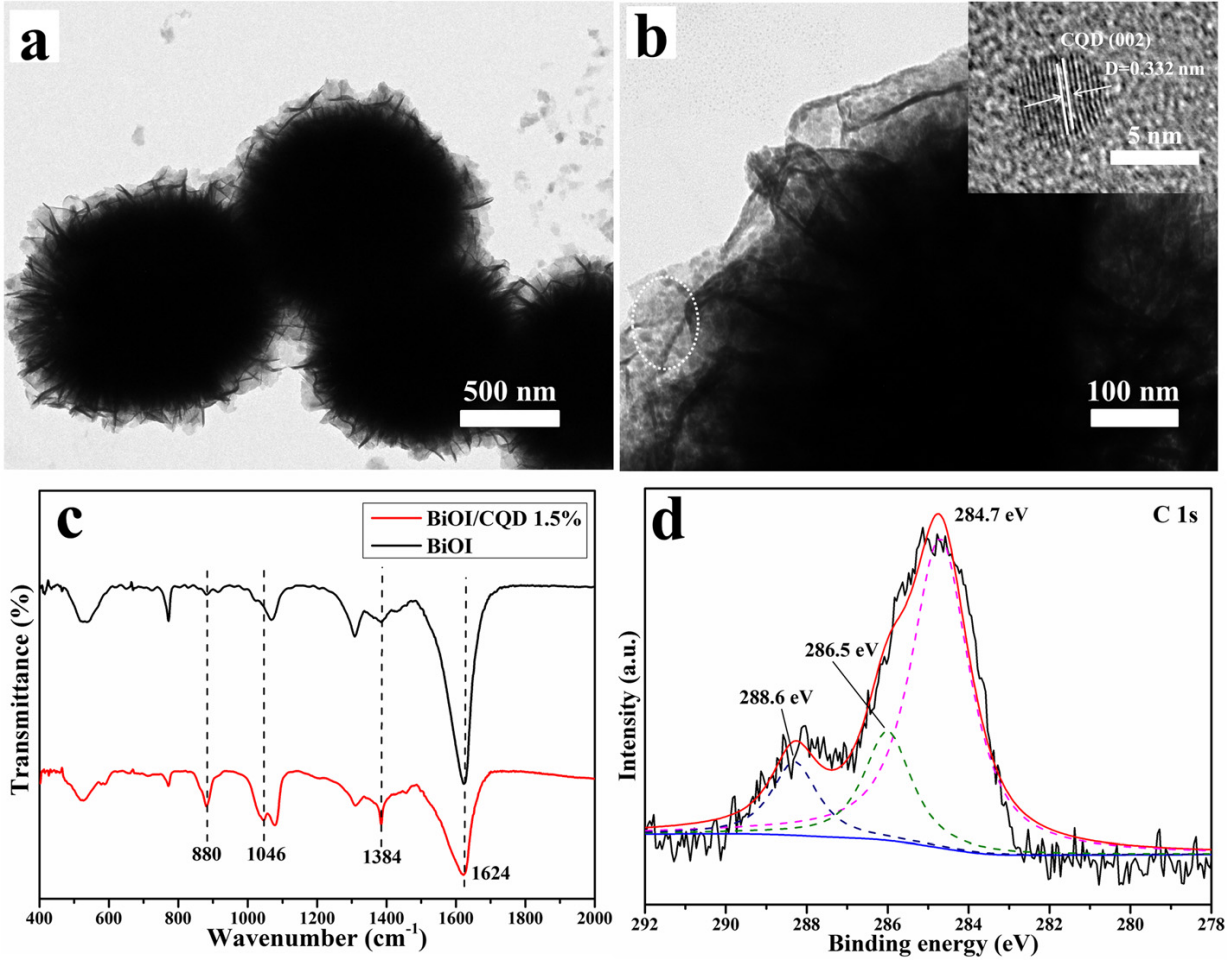
The TEM images of CQD/BiOI 1.5 wt.% sample (**a**, **b**). FT-IR spectra of pure BiOI and CQD/BiOI 1.5 wt.% samples (**c**). the XPS C 1s spectra of the CQD/BiOI 1.5 wt.% samples (**d**)

For a further investigation, the TEM and HRTEM were shown in Fig. 2a, b. Obviously, the nanoplates and microsphere morphology can be found in Fig. 2a, which was in agreement with the SEM result before. The high-resolution image was shown in Fig. 2b. Clearly, many uniformed particles were distributed on the surface of the BiOI; and in the corresponding HRTEM image, it can be seen that these particles have distinct lattice spacing. An atomic spacing (0.332 nm) could be distinguished, which could be ascribed to the (002) lattice fringes of CQD. The TEM and HRTEM results were directly indicated that the CQDs were successfully modified on the BiOI microsphere.

FT-IR spectra were also carried out to further characterization of CQD/BiOI (Fig. 2c). The absorption band at 1624 cm^−1^ should assign to the stretching modes of BiOI [12]. The absorption band was located at 1384 cm^−1^ which should be attributed to the stretching modes of NO_3_^−^ groups and C=C [18], and the band at 1046 and 880 cm^−1^ was associated with the skeletal vibration of sp^2^ and sp^3^ C–H and C–OH [12]. Obviously, due to the existence of CQDs, the bond was largely enhanced, which further demonstrated the existence of CQDs in these composites.

XPS spectrum was also used to study the surface properties of the CQD-modified BiOI sample as shown in Fig. 2d. It can be seen that C peaks were at 284.7, 286, and 288.3 eV, which could be assigned to the C–C bond with the sp^2^ orbital, C–O–C bond, and C=O bond, respectively [19]. As for the O 1s (Additional file 1: Figure S2a shown), two peaks located at 530.8 and 531.4 eV also should be ascribed to the C–O–C and C=O bond in CQDs, respectively. The Bi 4f and I 3d also were shown in Additional file 1: Figure S2b, c, both of which were consistent with the reported [18, 20].

**Fig. 3:**
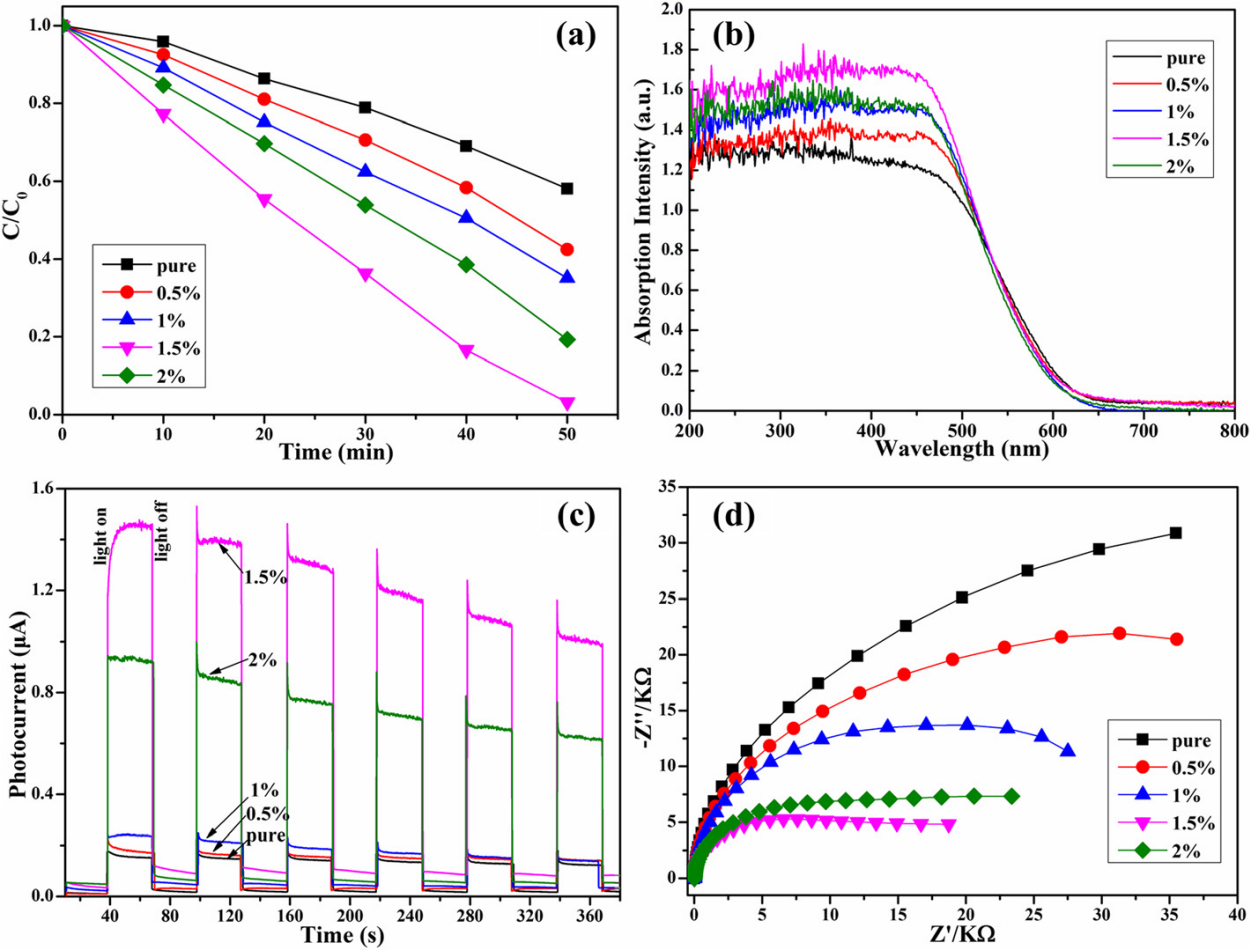
Photocatalytic degradation of MO in the presence of pure BiOI and CQDs/BiOI materials under visible light (*λ* > 420) irradiation (**a**). The UV-vis diffuse reflectance spectroscopy of the pure BiOI and the series of CQD/BiOI samples (**b**). Transient photocurrent responses (**c**) and electrochemical impedance spectroscopy (EIS) Nyquist plot (**d**) of the series samples

Before the photodegradation process, the adsorption–desorption property was tested during 60 min and the result was given in Additional file 1: Figure S3. It can be seen that all the samples shown excellent adsorption ability, which should be attributed to the huge specific surface areas of BiOI. This adsorption ability was enhanced with the CQD content increased, which should ascribe to the surface electron accumulated in CQDs [21]. The photocatalytic activities were evaluated as shown in Fig. 3a. Clearly, all the CQD-modified BiOI samples exhibited higher photocatalytic activity than pure BiOI, and the photocatalytic efficiency was 1.5 wt.% > 2 wt.% > 1 wt.% > 0.5 wt.% > pure BiOI. For the 1.5 wt.% sample, it can degrade 98 % of MO in 50 min while there is only 40 % of the pure BiOI sample. The CQDs would act as an electron-accepting and transport center, which would result in a lower recombination rate of photoinduced electron–hole pairs.

The light absorption and the charge transportation and separation were the key properties of the high performance of CQD/BiOI photocatalyst. UV-vis spectroscopy has been proved for understanding the electronic structure of semiconductors. As can be seen in Fig. 3b, the pure BiOI sample could absorb the wavelength less than 750 nm which indicate a strong light absorption and the result was in accordance with the previous report [4, 5]. Meanwhile, for the CQD-modified samples, the absorption intensity increased with the CQD content increase. The increased light absorption may generate more electron–hole pairs during the photocatalytic process.

As well known, the charge separation is the most complex and key factor essentially determining the efficiency of photocatalysis [22]. For a deep investigation, the PEC system was accompanied to investigate the photophysical behaviors of photogenerated electron–hole pairs as shown in Fig. 3c. It was found that the photocurrent response of all CQD/BiOI samples were higher than the pure BiOI sample, especially. As shown, the CQD/BiOI 1.5 wt.% sample was nearly seven times higher than the pure BiOI. The result suggested a more efficient separation and longer lifetimes of photoexcited electron–hole pairs. The EIS result (shown in Fig. 3d) reflected that the impedance arc radius of CQD/BiOI was smaller than the pure BiOI under visible light, indicating an enhanced separation efficiency of the photoexcited charge carriers in CQD/BiOI. In this regard, the transient photocurrent response and EIS results revealed an analogous trend with respect to the photocatalytic activity of the samples. Furthermore, the PL result also indicated that the CQD-modified BiOI could effectively decrease the recombination of the photoinduced electrons and holes (as seen in Additional file 1: Figure S4).

**Fig. 4:**
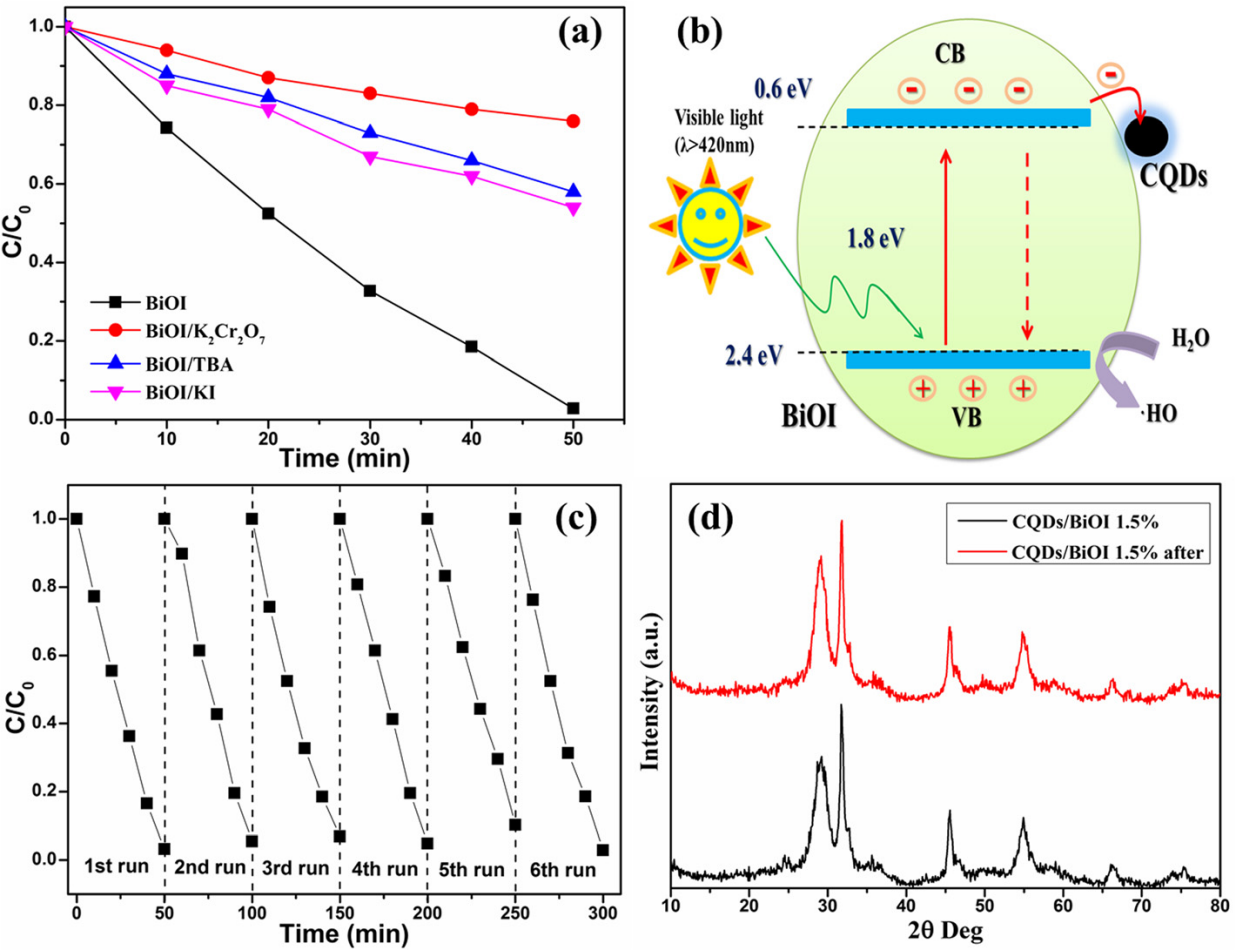
Photocatalytic activity comparison of the CQD/BiOI 1.5 wt.% sample in different photocatalysis systems under visible light irradiation (**a**). The schematic of the separation and transfer of photogenerated charges in the CQD/BiOI combined with the possible reaction mechanism of the photocatalytic procedure (**b**). Stability tests of CQD/BiOI 1.5 wt.% sample (**c**) and the XRD pattern of the sample before and after stability tests (**d**)

To determine the involvement of active radical species during photocatalysis, we performed a trapping experiment (Fig. 4a) for the detection of the hydroxyl radical (·OH), hole (h^+^), and electron (e^−^) in the photocatalytic process, taking the CQD/BiOI 1.5 wt.% sample as an example. The degradation behavior of MO is decreased upon the addition of TBA, K_2_Cr_2_O_7_, and KI, respectively, validating that ·OH radicals, photoexcited electrons, and h^+^ are the main active species for MO removal.

Based on the above results, the reaction mechanism diagram of CQD/BiOI photocatalysts was proposed as shown in Fig. 4b. The band gap of BiOI was about 1.8 eV, which can be easily excited by visible light. However, the *E*_CB_ and *E*_VB_ of BiOI were 0.6 and 2.4 eV, respectively. Hence, ·OH could not be produced via an e^−^ → ·O_2_^−^ → H_2_O_2_ → ·OH route. For the VB holes in BiOI, it can oxidize OH^−^ or H_2_O into ·OH due to their high potential energy; thus, it can be concluded that ·OH should be generated only via an h^+^ → OH^−^/H_2_O → ·OH route [18]. Furthermore, the photogenerated electrons would transfer to the CQDs due to their excellent electronic conductivity, which resulted in effective separation process for the photogenerated electron–hole pairs. The transferred electrons will accumulate on the CQDs and then inhibit the recombination of the electron–hole pairs. Obviously, the enhanced photocatalytic activity can be achieved, and the CQDs would play a crucial role in this process.

The photochemical and structural stability of a catalyst is important for practical applications. The stability of CQD/BiOI was tested by carrying out the photocatalytic reaction for multiple runs. The results were presented in Fig. 4c. The photocatalytic activity during the sixth runs can be observed. This result demonstrated that the CQD/BiOI composites have a stable photochemical property. Moreover, the almost unchanged XRD spectra of CQD/BiOI before and after the stability test (Fig. 4d) further indicated the phase stability of the CQD-modified BiOI photocatalysts.

## Conclusions

In conclusion, CQD-modified BiOI photocatalysts were synthesized using a facile hydrothermal treatment process. After being modified by CQDs, the photocatalytic activities of degradation of MO under visible light irradiation were increased greatly. The significant improvement in photocatalytic performance was attributed to the crucial role of CQDs in the samples. The CQD modification has several advantages, including enhanced light harvesting, improvement of interfacial charge transfer, and suppression of charge recombination. This work provides useful information on the design and fabrication of other CQD-modified semiconductor materials.

## Additional File

Additional file 1: Supplementary data associated with this article. (DOCX 17472 kb)

## References

[1] Fujishima A, Honda K (1972). Electrochemical photolysis of water at a semiconductor electrode. Nature.

[2] Wen Y, Liu BT, Zeng W, Wang YH (2013). Plasmonic photocatalysis properties of Au nanoparticles precipitated anatase/rutile mixed TiO_2_ nanotubes. Nanoscale.

[3] Liu BT, Tian LL, Wang YH (2013). One-pot solvothermal synthesis of ZnSe · xN_2_H_4_/GS and ZnSe/N-GS and enhanced visible-light photocatalysis. ACS Appl. Mater Interfaces.

[4] Di J, Xia JX, Ge YP, Li HP, Ji HY, Xu H, Zhang Q, Li HM, Li MN (2015). Novel visible-light-driven CQDs/Bi_2_WO_6_ hybrid materials with enhanced photocatalytic activity toward organic pollutants degradation and mechanism insight. Applied Catalysis B: Environmental.

[5] Han JL, Zhu GQ, Hojamberdiev M, Peng JH, Zhang X, Liu Y, Ge B, Liu P (2015). Rapid adsorption and photocatalytic activity for Rhodamine B and Cr(VI) by ultrathin BiOI nanosheets with highly exposed {001} facets. New J. Chem..

[6] Ou MY, Dong F, Zhang W, Wu ZB (2014). Efficient visible light photocatalytic oxidation of NO in air with band-gap tailored (BiO)_2_CO_3_–BiOI solid solutions. Chem. Eng. J..

[7] Li XY, Wang HQ, Shimizu Y, Pyatenko A, Kawaguchi K, Koshizaki N (2011). Preparation of carbon quantum dots with tunable photoluminescence by rapid laser passivation in ordinary organic solvents. Chem. Commun..

[8] Li H, He X, Liu Y, Huang H, Lian S, Lee ST, Kang Z (2011). One-step ultrasonic synthesis of water-soluble carbon nanoparticles with excellent photoluminescent properties. Carbon.

[9] Zhang YH, Tang ZR, Fu X, Xu YJ (2011). Engineering the unique 2D mat of graphene to achieve graphene-TiO_2_ nanocomposite for photocatalytic selective transformation: what advantage does graphene have over its forebear carbon nanotube?. ACS Nano.

[10] Zhang LM, Diao SO, Nie YF, Yan K, Liu N, B. Dai Y, Xie Q, Reina A, Kong J, Liu ZF (2011). Photocatalytic patterning and modification of grapheme. J. Am. Chem. Soc..

[11] Yu HJ, Zhao YF, Zhou C, Shang L, Peng Y, Cao YH, Wu LZ, Tung CH, Zhang TR (2014). Carbon quantum dots/TiO_2_ composites for efficient photocatalytic hydrogen evolution. J. Mater. Chem. A.

[12] Zhang HC, Huang H, Ming H, Li HT, Zhang LL, Liu Y, Kang ZH (2012). Carbon quantum dots/Ag_3_PO_4_ complex photocatalysts with enhanced photocatalytic activity and stability under visible light. J. Mater. Chem..

[13] Di J, Xia JX, Ji MX, Li HP, Xu H, Li HM, Chen R (2015). The synergistic role of carbon quantum dots for the improved photocatalytic performance of Bi_2_MoO_6_. Nanoscale.

[14] Zhu SJ, Meng QN, Wang L, Zhang JH, Song YB, Jin H, Zhang K, Sun HC, Wang HY, Yang B (2013). Highly photoluminescent carbon dots for multicolor patterning, sensors, and bioimaging. Angew. Chem. Int. Ed..

[15] Liu BT, Peng LL (2013). Facile formation of mixed phase porous TiO_2_ nanotubes and enhanced visible-light photocatalytic activity. Journal of Alloys and Compounds.

[16] Chen Y, Liu BT, Chen JF, Tian LL, Huang L, Tu M, Tan S (2015). Structure design and photocatalytic properties of one-dimensional SnO_2_-TiO_2_ composites. Nanoscale Research Letters.

[17] Tang D, Zhang HC, Huang H, Liu RH, Han YZ, Liu Y, Tong CY, Kang ZH (2013). Carbon quantum dots enhance the photocatalytic performance of BiVO_4_ with different exposed facets. Dalton Trans..

[18] Dong GH, Ho WK, Zhang LZ (2015). Photocatalytic NO removal on BiOI surface: the change from nonselective oxidation to selective oxidation. Applied Catalysis B: Environmental.

[19] Yu Y, Kwak SY (2012). Carbon quantum dots embedded with mesoporous hematite microsphere as efficient visible light-active photocatalysts. J. Mater. Chem.

[20] Gurunathan K (2004). Photocatalytic hydrogen production using transition metal ions-doped γ-Bi_2_O_3_ semiconductor particles. Int. J. Hydrogen Energy.

[21] Liu JC, Bai HW, Wang YJ, Liu ZY, Zhang XW, Sun DD (2010). Self-assembling TiO_2_ nanorods on large graphene oxide sheets at a two–phase interface and their Anti-recombination in photocatalytic applications. Adv. Funct. Mater..

[22] Di J, Xia JX, Ji MX, Wang B, Yin S, Zhang Q, Chen ZG, Li HM (2015). Carbon quantum dots modified BiOCl ultrathin nanosheets with enhanced molecular oxygen activation ability for broad spectrum photocatalytic properties and mechanism insight. ACS Appl. Mater. Interfaces.

