# Supplementary material for: Enhanced Photocatalytic Activity of the Carbon Quantum Dot-Modified BiOI Microsphere
Source: Nanoscale Res Lett. 2016 Feb 3;11:60. doi: 10.1186/s11671-016-1262-7 (PMC4740481; doi:10.1186/s11671-016-1262-7)
Supplement: Additional file 1: — Supplementary data associated with this article. (DOCX 17472 kb) [file 11671_2016_1262_MOESM1_ESM.docx]

Supplementary Information

**Enhance photocatalytic activity of the Carbon Quantum Dots modified BiOI microsphere**

Yuan Chen^1 2^, Qiuju Lu^1^, Xuelian Yan^1 2^, Qionghua Mo^1 3^, Yun Chen^1^, Bitao Liu^1 *^, Liumei Teng^1^, Wei Xiao^1 *^, Liangsheng Ge^1^, Qinyi Wang^4^.

^1^ Research Institute for New Materials Technology, Chongqing University of Arts and Sciences, Yongchuan Chongqing 402160, China.

^2^ School of material science and engineering, Chongqing University of Technology, Banan Chongqing 400054, China.

^3^ Faculty of Materials and Energy, Southwest University, Beibei Chongqing 400715, China.

^4^ Department of Chemical Engineering, University of Missouri, Columbia, MO, 65211-2200, United States.

^*^ Corresponding author. Tel.: +86-023-49891752.

E-mail: [liubitao007@163.com](mailto:liubitao007@163.com), [showame@aliyun.com](mailto:showame@aliyun.com).


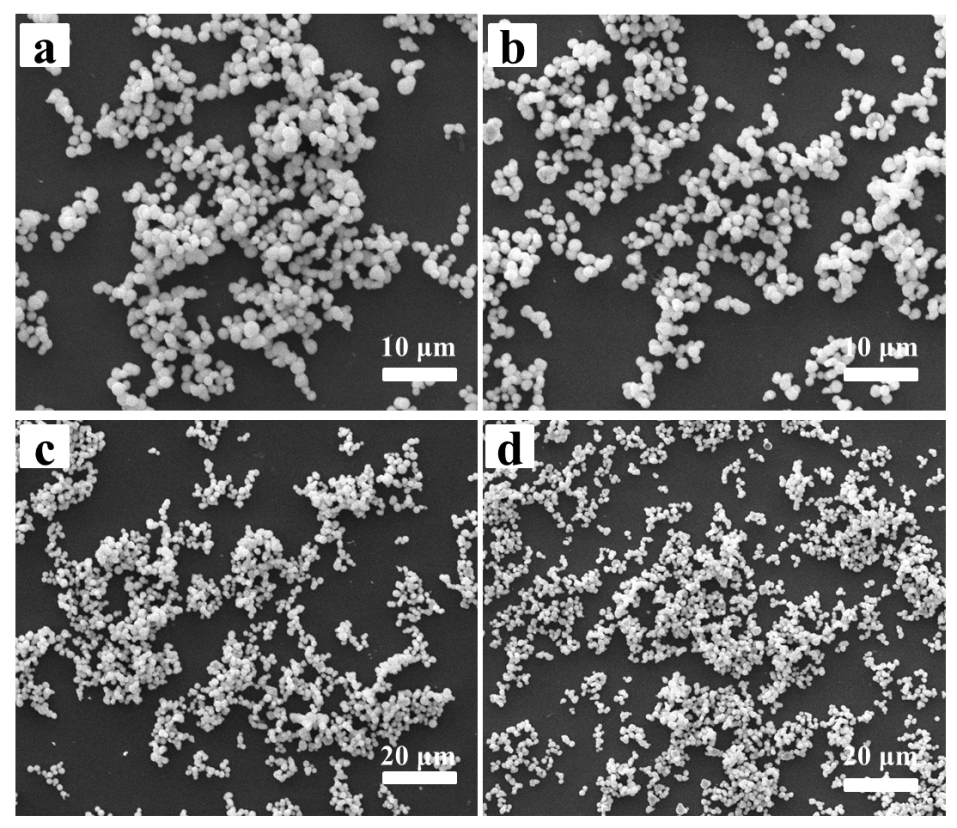


Figure S1. The SEM images of the series BiOI/CQDs composites: pure (a); 0.5% (b); 1% (c) and 2% (d).


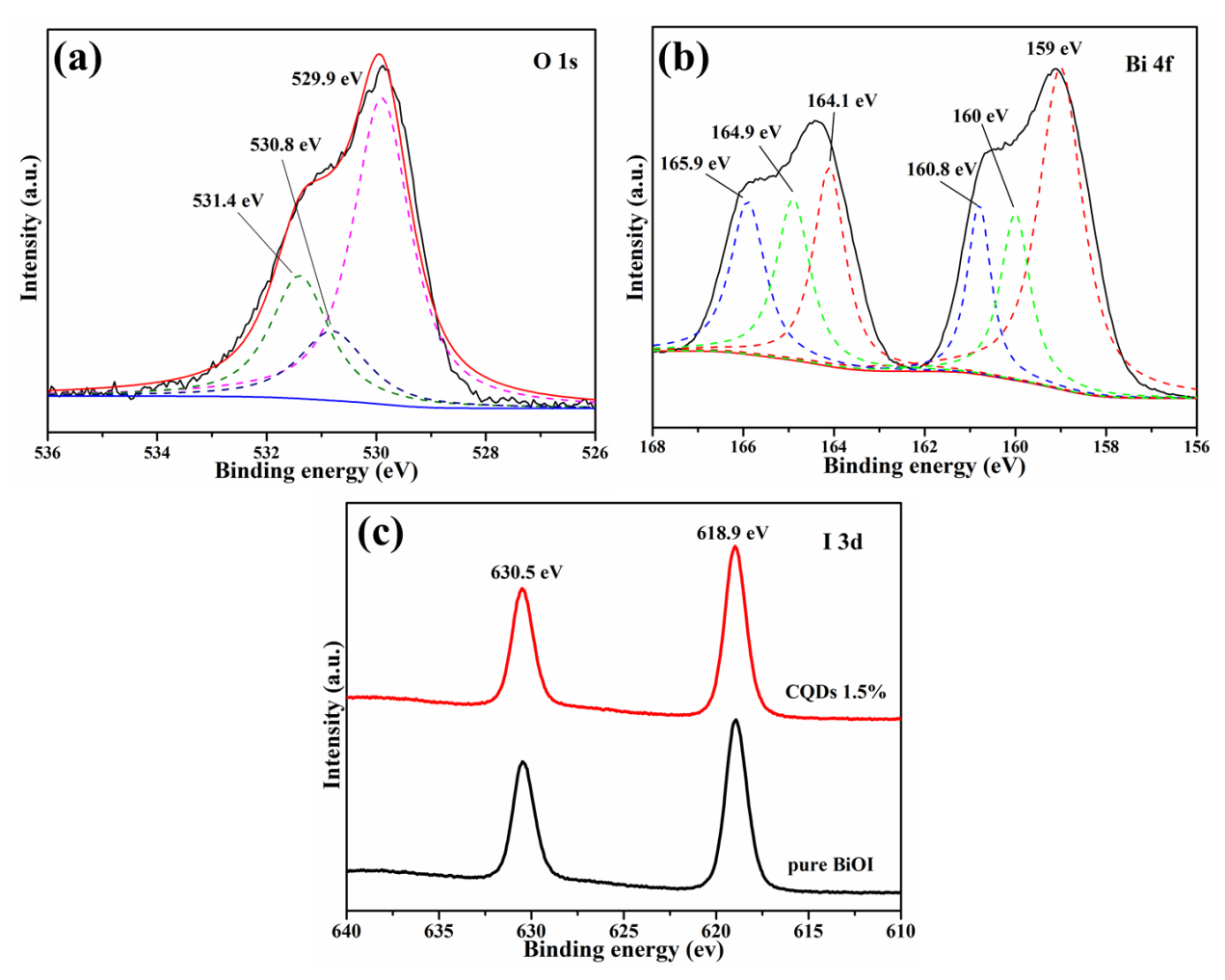


Figure S2. The XPS high resolution spectrum of the BiOI/CQDs 1.5% sample: O 1s (a); Bi 4f (b) and I 3d (c).





Figure S3. The adsorption-desorption curves of series BiOI/CQDs nanocomposites under 40 mg/L MO solution and dark surroundings (a); Time profile of MO absorbance spectra observed during incubation with CQDs/BiOI under visible light irradiation (b).





Fig S4. The PL spectra of BiOI and BiOI/CQDs 1.5wt% samples.
